# T cells accumulate in non-diabetic islets during ageing

**DOI:** 10.1186/s12979-021-00221-4

**Published:** 2021-02-23

**Authors:** Heather C. Denroche, Stéphanie Miard, Sandrine Sallé-Lefort, Frédéric Picard, C. Bruce Verchere

**Affiliations:** 1 Canucks for Kids Fund Childhood Diabetes Laboratories, BC Children’s Hospital Research Institute, Department of Surgery, University of British Columbia, Vancouver, British Columbia Canada; 2 Institut universitaire de cardiologie et de pneumologie de Québec, Université Laval, Québec, Québec Canada; 3 Faculté de pharmacie, Université Laval, Québec, Québec Canada; 4 Departments of Surgery and Pathology & Laboratory Medicine, BC Children’s Hospital Research Institute, Centre for Molecular Medicine and Therapeutics, University of British Columbia, 950 West 28th Ave, Vancouver, British Columbia V5Z 4H4 Canada

**Keywords:** T cells, Islets, Ageing, High fat diet, Insulin secretion, Mice, Human islets

## Abstract

**Background:**

The resident immune population of pancreatic islets has roles in islet development, beta cell physiology, and the pathology of diabetes. These roles have largely been attributed to islet macrophages, comprising 90% of islet immune cells (in the absence of islet autoimmunity), and, in the case of type 1 diabetes, to infiltrating autoreactive T cells. In adipose, tissue-resident and recruited T and B cells have been implicated in the development of insulin resistance during diet-induced obesity and ageing, but whether this is paralleled in the pancreatic islets is not known. Here, we investigated the non-macrophage component of resident islet immune cells in islets isolated from C57BL/6 J male mice during ageing (3 to 24 months of age) and following similar weight gain achieved by 12 weeks of 60% high fat diet. Immune cells were also examined by flow cytometry in cadaveric non-diabetic human islets.

**Results:**

Immune cells comprised 2.7 ± 1.3% of total islet cells in non-diabetic mouse islets, and 2.3 ± 1.7% of total islet cells in non-diabetic human islets. In 3-month old mice on standard diet, B and T cells each comprised approximately 2–4% of the total islet immune cell compartment, and approximately 0.1% of total islet cells. A similar amount of T cells were present in non-diabetic human islets. The majority of islet T cells expressed the αβ T cell receptor, and were comprised of CD8-positive, CD4-positive, and regulatory T cells, with a minor population of γδ T cells. Interestingly, the number of islet T cells increased linearly (R^2^ = 0.9902) with age from 0.10 ± 0.05% (3 months) to 0.38 ± 0.11% (24 months) of islet cells. This increase was uncoupled from body weight, and was not phenocopied by a degree similar weight gain induced by high fat diet in mice.

**Conclusions:**

This study reveals that T cells are a part of the normal islet immune population in mouse and human islets, and accumulate in islets during ageing in a body weight-independent manner. Though comprising only a small subset of the immune cells within islets, islet T cells may play a role in the physiology of islet ageing.

**Supplementary Information:**

The online version contains supplementary material available at 10.1186/s12979-021-00221-4.

## Background

Infiltration of pancreatic islets by immune cells, namely T cells, is a well known pathology of type 1 diabetes. More recently, islet inflammation has been recognized as a hallmark of type 2 diabetes [1–3], and is particularly induced by the presence of islet amyloid [4–9]. The interactions between immune cells and beta cells are not solely deleterious. Healthy, non-diabetic islets contain resident macrophages, making up ≥90% of islet immune cells, and are present in quantities of 2 to 13 macrophages per islet [10–14]. Islet macrophages play important roles in tissue homeostasis, islet development, beta cell regeneration, and physiological insulin secretion in mice [15–19]. Little is known, however, about the other cells comprising resident immune cells within islets, and whether these are altered in islets under various metabolic states.

T and B cells in adipose tissue have been implicated in modulating insulin sensitivity. In obesity, B2 cells and Th1-polarized T cells accumulate in visceral adipose tissue and contribute to insulin resistance, whereas B1, B-regulatory cells, Th2 and T-regulatory cells promote insulin sensitivity, and are reduced in number and/or function in obese visceral adipose tissue [20–28]. Ageing is also associated with inflammation in adipose tissue promoting insulin resistance, though the changes are distinct from those of obesity. T cells accumulate in aged visceral adipose tissue whereas the total number of macrophages is relatively unperturbed [21, 29–31], and unlike in diet-induced obesity, adipose tissue T regulatory cells accumulate and contribute to worsening glucose homeostasis during ageing [21, 29]. B2 cells also accumulate in white adipose tissue with age, and contribute to insulin resistance and glucose intolerance [32]. Despite the growing body of evidence that adipose-tissue T and B cells are important in glucose metabolism, the role of these cells in pancreatic islets during ageing and obesity is unclear. In this study, we aimed to investigate whether lymphocytes are present in non-diabetic pancreatic islets, and whether their numbers are altered by age or obesity.

## Results

### Glucose tolerance and insulin secretory capacity increase in advanced-age mice

**Fig. 1:**
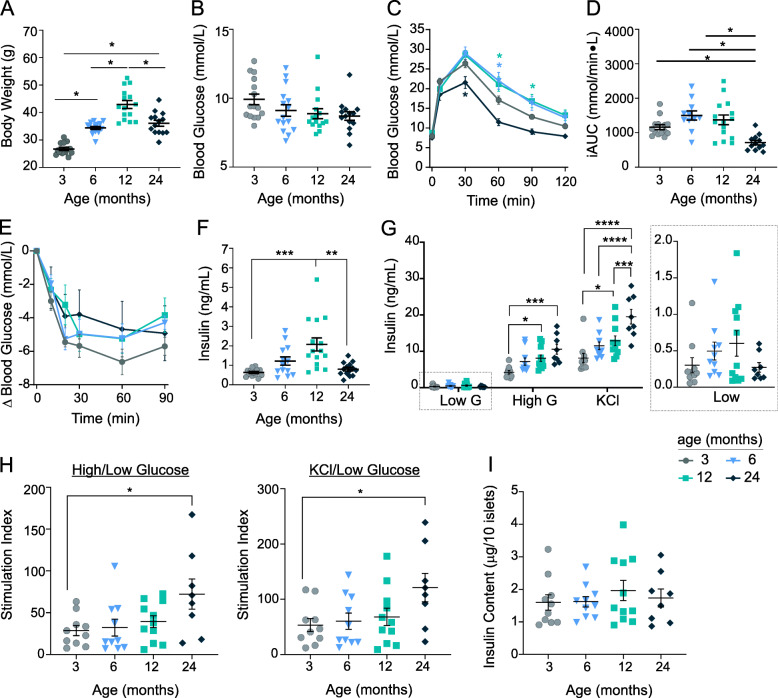
Metabolic characterization of aged mice. Metabolic measurements were performed in 3-, 6-, 12- and 24-month old C57BL/6 male mice. Non-fasted body weight (**a**) and blood glucose (**b**); glucose tolerance tests represented as glucose traces (**c**, *n* = 11–15) and incremental AUC (**d**); insulin tolerance tests normalized to baseline (**e**, *n* = 4–5), and 6-h fasted plasma insulin (**f**). **g**-**h** Insulin secretion from isolated islets, under low glucose (low G), high glucose (high G) and 30 mM KCl, represented as insulin concentration (**g**, with low G condition shown again on smaller scale on the right), and stimulation index relative to low glucose (**h**). Insulin content was measured following the final KCl stimulation (**i**). Data represent values for individual mice with mean ± SEM overlaid. Statistical analyses were performed via Kruskal-Wallis test with Dunn’s post-hoc test (**a**,**d**,**f**), or one-way ANOVA (B,G-I) or two-way repeated measures ANOVA (**c**,**e**) with Tukey’s post-hoc test (**b**,**c**,**e**,**g**-**i**). * *p* < 0.05*,* ** *p* < 0.01, *** *p* < 0.001, **** *p* < 0.0001 vs 3-month old unless other comparison specified

We first examined the impact of ageing from 3 to 24 months on glucose metabolism in male C57BL/6 J mice. Body weight increased with age up to 12 months, but was reduced in 24-month old mice (36.1 ± 4.1 g), to a level comparable to 6-month old mice (35.4 ± 2.1 g) (Fig. 1a). A decline in body weight at 2 years of age is consistent with previously published observations across many inbred mouse strains [33]. Non-fasted and 6-h fasted blood glucose levels were not altered by ageing (Fig. 1b,c). Ageing to 6 and 12 months corresponded with moderately impaired glucose tolerance relative to 3-month old mice (Fig. 1c-d), and ageing to 24 months substantially lowered glucose excursions compared to all other age groups, including 3-month old mice. Insulin tolerance tests indicated that insulin sensitivity cannot account for the improvement in glucose tolerance in 24-month old mice (Fig. 1e). Fasted plasma insulin levels increased with age up to approximately 2.7 fold at 12 months (Fig. 1f), consistent with previous observations [34, 35]. In 24-month old mice, fasted insulin levels were similar to those of 3-month old mice. Ex vivo insulin secretion from isolated islets in low glucose conditions tended to increase with age up to 12 months, and glucose- and KCl-stimulated insulin secretion increased with age, with 24-month old mice displaying the highest stimulated responses compared to all other age groups (Fig. 1g,h). Islet insulin content was not altered in aged mice (Fig. 1i). The increased glucose- and KCl- stimulated insulin secretion in 24-month old mice suggests that stimulus-coupled insulin secretion is augmented with age, potentially accounting for improved glucose tolerance in advanced-aged mice, and supports previous findings that aged mice have improved glucose tolerance and increased insulin secretion independent of insulin content [34–37].

### T cells accumulate in ageing islets

**Fig. 2:**
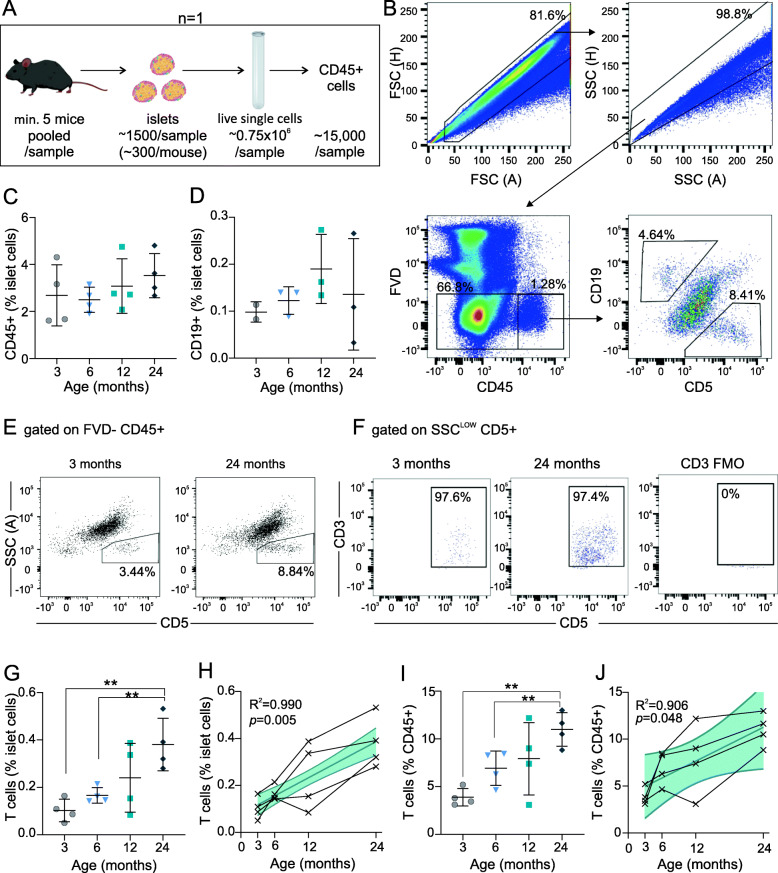
Characterization of mouse islet immune cells during ageing. Islets were isolated from C57Bl/6 J male mice aged 3, 6, 12 and 24 months for analysis by flow cytometry. **a** Schematic of flow cytometry protocol; islets from a minimum of 5 mice were pooled per sample to obtain sufficient dispersed islet CD45+ cells for analysis. Each pooled sample of ≥5 mice is considered one individual biological sample (*n* = 1). **b** Cells were gated on forward-scatter (FSC), side-scatter (SSC), viability (FVD-), CD45+, and subsequently CD19 or CD5. Data are from a representative 6-month old mouse islet sample. **c**-**d** CD45+ and CD19+ cells expressed as a percent of total viable islet cells. **e** Representative plots of SSC^low^CD5+ populations from 3-month and 24-month old mouse islets. **f** Cells gated on SSC^low^CD5+ are positive for CD3+, box represents CD3 + CD5+ cells. **g**-**j** T cells expressed as a percent of total viable islet cells (G-H) or CD45+ islet cells (**i**-**j**). Linear regression (**h**,**j**) analysis shows 95% confidence interval in blue and Pearson correlation coefficient, connecting lines between data points are from a given cohort of mice run in one independent experiment. Data represent independent biological samples, each run as an independent experiment, with mean ± SD overlaid (**c**,**d**,**g**-**j**), and were analyzed by one way ANOVA with Dunnett’s T3 multiple comparisons test (**c**,**g**,**i**) or Kruskal-Wallis test with Dunn’s multiple comparisons test (**d**). * *p* < 0.05, ** *p* < 0.01, *** *p* < 0.001, **** *p* < 0.0001. Numbers in FACS plots represent the percent of cells in each selection as a function of the parent population

We next assessed the effect of ageing on islet-resident immune populations. Islets from C57BL/6 J mice of the four age groups were isolated and hand-picked to purity. Islets from a minimum of five mice were pooled per sample, to obtain a sufficient number of islet immune cells, and dispersed into single-cell suspensions for analysis by flow cytometry (Fig. 2a). Islet cells were gated on singlets and viability prior to analysis of immune cell populations (Fig. 2b). Immune cells (CD45+) accounted for 2.7 ± 1.3% of viable islet cells in 3-month old mice to 3.5 ± 0.9% in 24-month old mice (Fig. 2c). As we were initially interested in resident islet B-cell populations, islet cells were additionally stained for CD19, along with CD23 and CD5 to differentiate B-cell subsets. Approximately 90% of islet immune cells were negative for CD19 and CD5 (Fig. 2b), consistent with reports that the vast majority of resident islet immune cells are macrophages [10, 11, 38]. CD19+ cells were negative for CD5, consistent with B2 cells (Fig. 2b), and present at a frequency of approximately 0.1% of islet cells, which was not altered by age (Fig. 2d).

Surprisingly, in addition to B cells, we observed that islets contained a distinct population of CD19-CD5+ cells (Fig. 2b). CD19-CD5+ cells comprised 3.9 ± 0.9% of islet immune cells in 3-month old mice, had a side scatter profile (SSC^low^) consistent with lymphocytes, and accumulated with age (Fig. 2e-g). As CD5 expression is limited to B1a cells and T cells, we suspected that these were islet-resident T cells. Indeed, CD19-CD5+ cells expressed the T cell co-receptor, CD3, in all age groups (Fig. 2f). Islet T cells showed a positive linear correlation with age, comprising 0.10 ± 0.05% of islet cells at 3 months and rising approximately 4-fold to 0.38 ± 0.11% of islet cells at 24 months of age (R^2^ = 0.9902, *p* = 0.0049) (Fig. 2g-h). This increase was specific to T cells, and the frequency of T cells relative to islet immune cells increased with age from 3.9 ± 0.9% of CD45+ cells to 11.0 ± 1.8% of CD45+ cells (R^2^ = 0.9059, *p* = 0.048) (Fig. 2i-j).

**Fig. 3:**
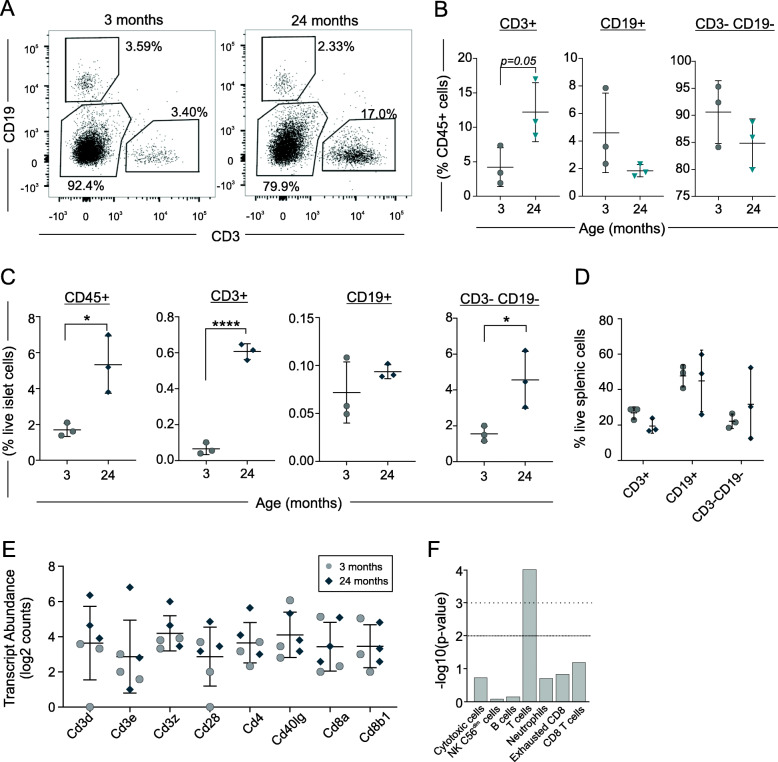
Islet T cells accumulate during ageing in non-diabetic mice. **a**-**c** Islets were isolated from 3- and 24-month old mice. Data represent 3 samples per age group, with islets from 5 to 7 mice pooled per sample. **a** Islet cells gated on FSC, SSC, viability (7AAD-) and CD45+, representative of 3 independent samples. **b**-**c** Quantification of immune cell subsets as a percent of immune (CD45+) cells (**b**) and total live cells (**c**) in islets from 3- and 24-month old mice, data represent mean ± SD, and were analyzed by unpaired T-test. **d** Quantification of immune cell subsets as a percent of total live cells in spleens from 3- and 24-month old mice, *n* = 3, gated on FSC, SSC, viability (7AAD-) and CD45+. **e** Transcript abundance of sorted CD3+ islet cells, in 3-month samples (black) and 24-month samples (blue), data represent mean ± SD. **f** Cell profile analysis was performed on transcriptome profiles obtained from sorted CD3+ islet cells from 3- and 24-month old mice via NSolver4.0, revealing a statistically significant T cell profile relative to other cell types, n = 3 samples, 5–7 mice pooled per sample. Numbers in FACS plots represent the percent of cells in each selection as a function of the parent population. * p < 0.05, ** p < 0.01, *** *p* < 0.001, **** *p* < 0.0001

We next assessed the characteristics of islet T cells by FACS sorting islet cells from an additional cohort of 3- and 24- month old mice. Single, viable islet cells were gated on CD45 and subsequently on CD19 and CD3 staining (Fig. 3a). In this experiment CD45+ cells increased with age (Fig. 3c), though this was not observed in the previous experiment. This was driven largely by an increase in CD3-CD19- cells which constitute the majority of CD45+ cells (Fig. 3c), and express CD11b (Supplemental Fig. 1), consistent with islet macrophages. However, in proportion to total CD45+ cells, there was a trend towards decreased CD3-CD19- and CD19+ cells with age and an increase in CD3+ cells (4.2 ± 2.8% vs 12.2 ± 4.3% of CD45+ cells in 3 and 24 month old mice) (Fig. 3b). This translated to an  increase in CD3+ cells from 0.08 ± 0.03% to 0.61 ± 0.08% of islet cells (Fig. 3c). There was no increase in CD3+ cells in the spleens of aged mice, consistent with previous reports [30] (Fig. 3d). We confirmed the expression of T cell transcripts in sorted islet CD3+ cells, and cell profile analysis (Nsolver4.0) confirmed a statistically significant T cell transcript profile (Fig. 3e,f).

**Fig. 4:**
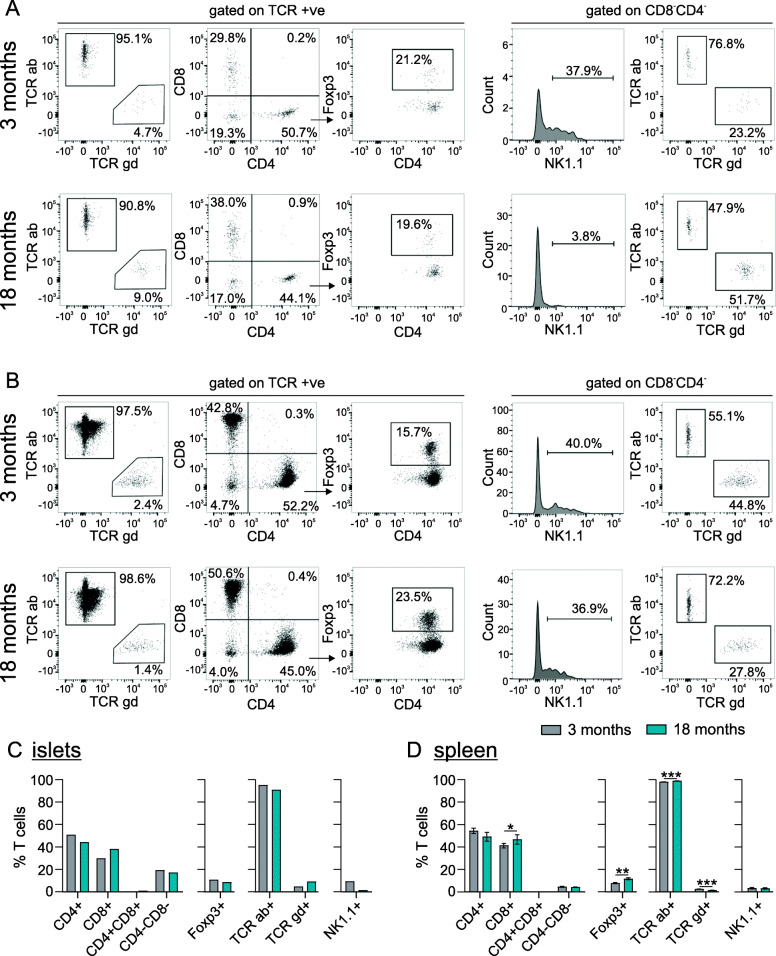
T cell subsets in islets of young and aged mice. Islets (**a**,**c**) and spleens (**b**,**d**) were isolated from 3- and 18-month old mice. Islets from 10 mice pooled per sample, n = 1 sample/age group and spleens from 3 individual mice were assessed per age group. Cells were assessed for surface markers TCR αβ (TCR ab), TCR γδ (TCR gd), CD4, CD8 and NK1.1 after gating on FSC, SSC, viability (7AAD-), CD45+ and TCR+ population. **a**-**b**) Numbers in FACS plots represent the percent of cells in each selection as a function of the parent population. **c**-**d** Quantification as a percent of total TCR+ cells shown as mean ± SD for islet (**c**) and spleen (**d**). Statistical analyses were performed via unpaired T-test with Welch’s correction (Foxp3+ and NK1.1+) or two-way ANOVA with Sidak multiple comparison test (CD4,CD8,TCR ab, TCR gd), * *p* < 0.05, ** p < 0.01, *** *p* < 0.001

We subsequently examined islet T cell subsets from young (3 month old) and advanced-aged (18 month old) mice by flow cytometry (Fig. 4a,c). The majority of islet T cells expressed the αβ T cell receptor (TCR) in both age groups. However, there was a small population of γδ TCR expressing cells, that increased from 4.7% of T cells at 3 months to 9.0% at 18 months of age. Islet T cells contained a mix of CD8+  and CD4+ cells, the frequencies of which were not robustly altered with age, consistent with the lack of an ageing effect on Cd4 and Cd8 transcripts in sorted islet T cells (Fig. 3e). The proportion of regulatory T cells (Foxp3+) was relatively constant in islets at ~ 20% of CD4+ cells across age groups (Fig. 4a,c). Similar proportions of CD4+, CD8+ and Foxp3+ cells were found in islets of 10-month old mice (Supplemental Fig. 1). There were very few CD4+CD8+ double positive cells at either age, however islets did contain an appreciable population of CD4-CD8- double negative cells, at 19.3 and 17.0% of T cells in 3- and 18-month old mice, respectively. The γδ T cell subset discussed above constituted a portion of CD4-CD8- cells; from 23.2% to 51.7% of CD4-CD8- cells in 3- and 18-month old mice, respectively. Interestingly, in 3-month old mice, 37.9% of CD4-CD8- cells expressed NK1.1, consistent with NKT cells, whereas only 3.8% of the double negative cells in 18-month old mice expressed NK1.1. The T cell subsets found in islets were distinct from those in the spleen of the same animals (Fig. 4b,d). γδ T cells were more rare and, in contrast to islets, decreased modestly from 2.4 to 1.4% with age, which has been previously reported [39]. Also unlike in islets, regulatory T cells increased modestly with age, while the NKT cell fraction was unaltered.

### T cells are present in non-diabetic human islets

**Fig. 5:**
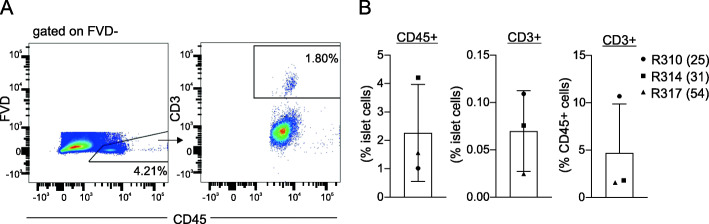
T cells are present in non-diabetic human islets. Islets from 3 non-diabetic, cadaveric human donors were dispersed for analysis. Cells were gated on FSC, SSC and viability (FVD-). **a** Representative data showing CD45+ cells as a function of viable islet cells (left) and CD3+ cells as a function of CD45+ cells (right). **b** Quantification of islet immune populations from the 3 samples, shown as mean ± SD. Each symbol corresponds to an individual donor, with age of donor in brackets. Numbers in FACS plots represent the percent of cells in each selection as a function of the parent population

To determine whether the presence of T cells in non-diabetic islets was generalizable to humans, we performed flow cytometric analysis on human islets from 3 cadaveric, non-diabetic human donors of ages 25, 30 and 54 years (Supplemental Table 2). Similar to mice, all human islet preparations contained a small proportion of CD45+ cells, comprising approximately 2.3 ± 1.7% of islet cells (Fig. 5a,b). Furthermore, all samples contained a population of CD3+ cells within pancreatic islets, at a frequency of 0.070 ± 0.043% of total live islet cells (Fig. 5a,b), comparable to frequencies we observed in young adult mice. There was no apparent correlation between age of donor and CD3+ or CD45+ cell frequency, however determinations of this manner would require a greater sample size.

### Islet T cells are not increased by comparable weight gain on high fat diet

**Fig. 6:**
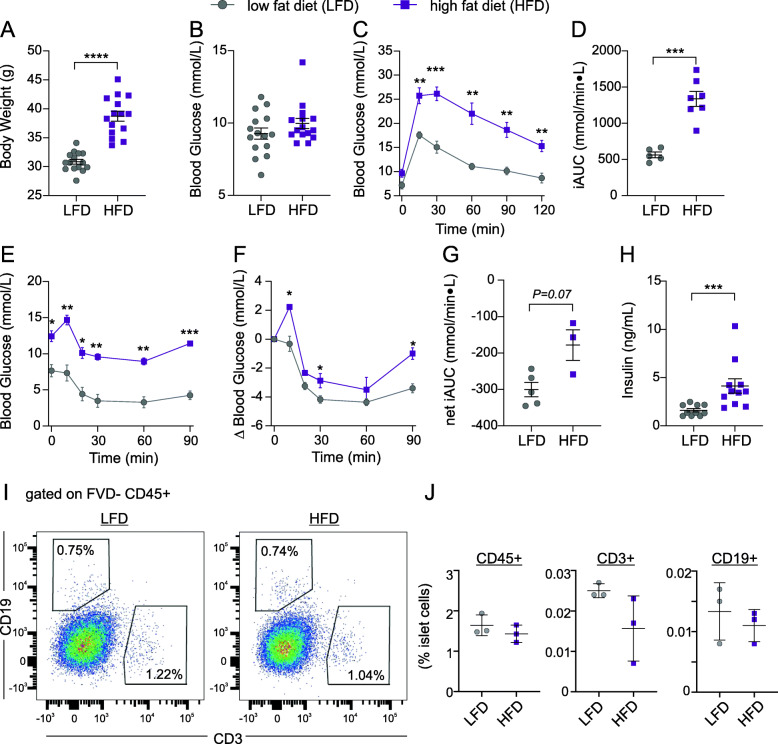
Weight gain via high fat feeding does not elicit T cell accumulation in islets. C57BL/6 J mice were fed a high fat diet (HFD, purple squares) or low fat diet (LFD, grey circles) for 9–12 weeks from 6 weeks of age. Non-fasted body weight (**a**) and blood glucose (**b**) after 9 weeks of diet. Glucose tolerance tests (**c**-**d**, *n* = 5–7), shown as glucose traces (**c**) and incremental AUC (**d**), and insulin tolerance tests (**e**-**g**, *n* = 3–5) at 10 and 11 weeks of diet, shown as raw blood glucose (**e**), delta blood glucose (**f**), and net AUC relative to baseline (**g**). **h** Fasted plasma insulin at 11–12 weeks of diet. Data represent mean ± SEM, and were analyzed by Student’s T-test with Welch’s correction (**a**,**d**,**g**), Mann-Whitney test (**b**,**h**), or two-way repeated measures ANOVA with Sidak’s multiple comparisons test (**c**,**e**,**f**). **i**-**j** Islets were isolated from LFD- and HFD-fed mice after 12 weeks of diet (*n* = 3 samples, 5 mice pooled per sample), dispersed, and gated on FSC, SSC, viability (7AAD-) and CD45+, and subsequently CD19 and CD3. **i** Representative data for LFD- and HFD-mouse islets. **j** Quantification of islet immune cell populations as a function of viable islet cells, data represent mean ± SD and were analyzed by Mann-Whitney test. Numbers in FACS plots represent the percent of cells in each selection as a function of the parent population

To determine whether the accumulation of intra-islet T cells was specific to ageing, or associated generally with obesity or increasing body weight, we next examined islet T cells in mice that gained a similar amount of weight through high fat diet (HFD) rather than ageing. Mice were HFD-fed for 12 weeks (up to 4 months of age) and compared to age-matched, low fat diet (LFD)-fed mice. This duration of HFD increased body weight by 25% relative to LFD-fed controls (39 ± 3 vs 31 ± 2 g) (Fig. 6a), a comparable increase to the ~ 28% increase observed in 6-month relative to 3-month old mice (34 ± 2 g vs 27 ± 2 g) (Fig. 1a). In terms of absolute body weight, HFD-fed mice were in the range of 12-month (43 ± 6 g) and 24-month (36 ± 4 g) old aged-mice (Fig. 1a). Basal blood glucose levels were not significantly altered by HFD relative to LFD (Fig. 6b), though glucose tolerance was impaired, as expected (Fig. 6c-d). HFD-fed mice were also insulin resistant compared to LFD-fed controls (Fig. 6e-g), and had a ~ 2.6 fold increase in plasma insulin levels (Fig. 6h), similar to the ~ 2.7 fold increase in insulin in 12-month relative to 3-month old mice (Fig. 1f). Thus, HFD achieved similar increases in body weight and insulin levels to 12 months of ageing but resulted in a more dramatic impairment in glucose tolerance. T and B cells were present in islets at frequencies of ~ 1% of immune cells in LFD-control mice, comparable to frequencies in 3-month old mice (Fig. 6i-j). Despite comparable increases in body weight and insulin to 12 months of ageing, HFD-fed mice showed no increase in islet CD3+ cells. The frequency of CD45+ cells in islets, as well as the number of CD19-CD3- cells was also unaffected by 12 weeks of high fat diet (Fig. 6j). Collectively, these data show that T cells are present in islets in the absence of autoimmune diabetes, and that there is a distinct increase in islet T cells during ageing that cannot be accounted for by weight gain.

## Discussion

In this study we found that T cells contribute to the normal islet immune cell repertoire in non-diabetic mice and humans. While macrophages comprise the majority of islet immune cells [10–14, 40], B and T cells each comprise up to approximately 5% of islet immune cells in young adult mouse islets. We also found that islet T cells accumulated within islets during ageing in mice. Islet T cells increased by 4- to 10-fold from 3 to 24 months of age, and this increase was specific to T cells. Though in one cohort of mice we observed an overall increase in islet CD45+ cells as well, this was not reproduced across independent experiments. Greater than 90% of islet T cells were αβ T cells, and these largely accounted for the increase in T cells with age, though there was also an increase in a minor population of γδ T cells. Islet T cells were comprised of a mixture of CD4+, CD8+ and FoxP3+ subsets that remained relatively constant with age. Interestingly, NKT cells were also present as a minor population in young islets and declined with age. An age-related accumulation of T cells was not observed in spleen, suggesting this phenomenon is islet-specific. In addition, the composition of T cell subsets present within islets appeared distinct from those in the spleen. Finally, two lines of evidence revealed islet T cell accumulation was uncoupled from body weight: T cell frequency progressively increased with age despite a modest decline in body weight in 24 month old mice; and islet T cell accumulation was not induced by HFD in young mice. Thus, we postulate that T cells play a role in islet biology (in the absence of autoimmune diabetes), and particularly, in adaptive changes to the pancreatic islet during ageing, though a functional role for islet T cells was not tested in this study.

Our findings contrast some studies that have claimed a lack of T cells in non-diabetic mouse islets. Though scarce relative to islet macrophages, our study clearly shows a small, yet persistent population of T cells in both mouse and human islets; this is consistent with a small number of studies that have reported T cells in non-diabetic islets [2, 41–43], but currently the contribution of this population to resident islet immune cells is not widely recognized. At approximately 0.1% of islet cells in young adult mice, there would be approximately one T cell present in an islet comprised of 1000 cells, which would increase to approximately 4 to 8 T cells in a similar sized islet in 2-year old mice. This does not simply reflect the expected increase in islet size or the number of total islet cells with age, as the increase is observed as a percent of total islet cells. Despite the paucity of T cells within pancreatic islets, these cells may still produce sufficient levels of local cytokines within the islet to influence the islet environment. Such is the case for islet macrophages, comprising only 2–10 cells per islet, which are the major source of islet IL-1β [6, 15], and play key roles in islet development and beta-cell adaptation and expansion [15–19, 40, 44]. Islet-resident group 2 innate lymphoid cells, another rare immune population in islets, have also been shown to influence insulin secretion, indirectly via islet macrophages [42]. These examples underline how a small number of cells can have a substantial impact on islet physiology.

The shift in the islet immune cell compartment in favour of T cells suggests these cells may play a specific ageing-related role in pancreatic islets. Interestingly, our data show that no single T cell subpopulation drives the increase in islet T cells. Rather, the relative proportion of T cell subsets remain stable, with the exception of an increase in the minor population of γδ T cells. Furthermore, the ageing-induced accumulation of islet T cells is not recapitulated by a similar degree of weight gain in HFD-fed mice, and thus is not driven by obesity per se. This parallels the divergent immune cell profiles in adipose tissue of aged versus obese mice [31]. Notably, the metabolic outcomes of ageing and diet-induced obesity in mice are different, with ageing resulting in increased insulin secretion and ultimately improved glucose tolerance in advanced age mice, whereas diet-induced obesity progressively impairs insulin secretion and glucose tolerance. Thus, the immunologic and metabolic state of ageing is distinct from that of obesity, and it is plausible that, like in adipose tissue, T cell accumulation in islets contributes to the regulation of glucose homeostasis during ageing.

The function and source of islet T cells during ageing, was not examined in this study due to the large number of mice that would be required, but warrants further investigation. The fact that T cell subsets in islets are distinct from the spleen suggest they are not at equilibrium with systemic ageing-induced changes in T cell populations. These T cells may reside long-term and expand in situ as has been shown for tissue resident memory T cells in skin [45], or they could be recruited to islets throughout ageing by secreted factors or the accumulation of an antigen target. In the future, it would be interesting to compare T cell populations in the islets to the exocrine pancreas during ageing, as this could be a source of islet T cells, as was recently suggested in type 1 diabetes [46]. The correlation between the progressive increase in insulin secretion during ageing, observed in this study and others [34–37], and the progressive accumulation of islet T cells reported here, is intriguing. Interestingly, a beta-cell supportive role of T cells has been reported in pre-diabetic NOD mice, in which the autoreactive T cells that home to islets have been shown to promote beta cell proliferation through production of cytokines IL-2, IL-6, IL-10, CCL3, and CCL5 [47]. In other organs, senescent cells attract innate and adaptive immune cells including T cells through secreted factors, and immune cells play an important role in senescent-cell clearance [48]. The replicatively-senescent beta cells that accumulate during ageing have a distinct profile from stress-induced senescent beta cells in obesity and diabetes [36, 49, 50], and may account for the unique immune profile of ageing islets. The function and source of the minor population of islet γδ T cells during ageing would also be interesting to pursue in future studies; γδ T cells are involved in the pathology of ageing [39], and have been reported in NOD mouse islets as playing a major role in diabetes pathology [51]. It is currently unclear if our findings are generalizable to humans; unlike in mice, insulin secretion in humans declines with advanced age [52], and future studies with a larger sample size will be needed to determine whether T cells accumulate in human islets during ageing.

## Conclusions

Collectively, this study demonstrates that T cells are part of the normal immune population of pancreatic islets in non-diabetic mice and humans, and that their numbers accumulate during ageing in mice. The source, function and target(s) of these T cells in non-diabetic islets, particularly during ageing, is an interesting avenue of future study.

## Methods

### Animals

Male C57BL/6 J mice were obtained from Jackson Laboratories (Strain #000664, Jackson Laboratories, Bar Harbor, ME) and aged up to 24 months at the Institut universitaire de cardiologie et de pneumologie de Québec-Université Laval (Québec, Canada) as part of the ageing mice colony of the Québec Network for Research on Ageing (RQRV). Mice were housed under 12-h light 12-h dark conditions at room temperature, with ad libitum access to regular chow (NIH-31 Teklad #7917, Envigo) and water. Mice were transported at 3, 6, 12 or 24 months to BC Children’s Hospital Research Institute (Vancouver, Canada). For analysis of T cell subsets in Fig. 4 only, pre-aged C57BL/6 J mice were purchased directly from Jackson Laboratories (##000664). All animal experiments were conducted at BC Children’s Hospital Research Institute where mice were housed under 12-h light 12-h dark conditions at room temperature, with ad libitum access to chow (Teklad #2918, Envigo) and water. Mice were acclimated for one week following arrival prior to any experimentation. High fat diet fed mice (Jackson Laboratories, cat#380050) were fed a 60% fat diet (#D12492, Research Diets, New Brunswick, USA) from 6 weeks of age. Chow-fed controls (Jackson Laboratories, cat#380056) were fed 10% fat diet (#D12450B, Research Diets) from 6 weeks of age.

### Metabolic tests

Blood glucose was measured via tail poke and measured with a OneTouch Ultra Glucometer (Life Scan Inc., Burnaby, Canada). For glucose tolerance tests, mice were fasted for 6 h and subsequently injected intraperitoneally with 2 g/kg (ageing study) or 1.5 g/kg body weight (diet study) glucose, and blood glucose measured at indicated time points as above. Blood was collected from the saphenous vein after a 6-h fast for the measurement of plasma insulin via Stellux Chemiluminescent Rodent Insulin ELISA (#80-INSMR-CH01, Alpco, Salem). For insulin tolerance tests, insulin (Novolin GE Toronto, Novo Nordisk) was injected at the 0.65 U/kg (ageing study) or 0.35 U/kg (diet study) and blood glucose measured at indicated time points.

### Flow cytometry and cell sorting

Islets were isolated via collagenase injection into the pancreatic duct, and dispersed for flow cytometry as previously published [10]. Briefly, islets were harvested from mice and hand-picked to purity, and subsequently exocrine-free islets were selected and dispersed with 0.02% trypsin for 3 min at 37 °C. Dispersions were stopped by addition of 7 mL of FACS buffer containing 2% FBS on ice. Dispersed cells were incubated with FcR block (Thermo Fisher Scientific), 10 min prior to addition of antibodies (Supplemental Table 1) for 30 min. 7-AAD or fixable viability dye eFluor780 (Thermo Fisher Scientific) served as a live-dead stain. Flow cytometry was conducted on a LSRFortessa (BD Biosciences, San Jose, USA) or BD LSR II (BD Biosciences). Cells were sorted on a BD FACS ARIA II (BD Biosciences) into PBS containing 2% FBS.

### Transcript analysis

FACS-sorted islet cells were washed, and lysed in a minimum of 5 μL Cells-to-CT buffer containing 1% DNAse I (Taqman Gene Expression Cells-to-CT Kit, Thermo Fisher Scientific), or a volume adjusted for a final cell concentration of ~ 1000 cells/μL for 5 min at RT, and stopped by addition of 10% Stop Solution, according to manufacturer instructions. Transcriptome analysis was performed via Nanostring nCounter XT (Nanostring Technologies, Seattle, USA) with the XT_PGX_MmV1_Immunology Code Set. Due to low cell input from 3-month old islets, pre-amplification of RNA was performed via Nanostring Low RNA Input Kit following manufacturer instructions. Briefly, RNA was diluted 1:2 in water, and 4 μL sample was reverse transcribed, followed by 8 rounds of amplification with M Immunology v1 Primers. All other samples were assessed without pre-amplification. Cell lysate or pre-amplified cDNA (5 μL) was added to hybridization mixtures for 18 h at 65 °C with a 70 °C-heated lid. Gene expression was measured with a Nanostring SPRINT Profiler, and data were analyzed via Nsolver 4.0 software.

### Glucose-stimulated insulin secretion

Duplicates of 10 size-matched islets per mouse were rested in Krebs-Ringer Bicarbonate buffer (129 mmol/L NaCl, 4.8 mmol/L KCl, 1.2 mmol/L MgSO_4_, 1.2 mmol/L KH_2_PO_4_, 2.5 mmol/L CaCl_2_, 5 mmol/L NaHCO_3_, 10 mmol/L HEPES, 0.5% bovine serum albumin) containing 2.8 mM glucose for 1 h at 37 °C, and were sequentially incubated for 45 min in KRB containing 2.8 mM (low) glucose, then 16.7 mmol/L (high) glucose, and then 30 mmol/L KCl (103.8 mmol/L NaCl, 30 mmol/L KCl, 1.2 mmol/L MgSO_4_, 1.2 mmol/L KH_2_PO_4_, 2.5 mmol/L CaCl_2_, 5 mmol/L NaHCO_3_, 10 mmol/L HEPES, 0.5% bovine serum albumin, 2.8 mmol/L glucose). Cell supernatants were collected for measurement of insulin secretion, and islets were lysed in acid:ethanol for insulin content. Insulin was measured via Chemiluminescent Rodent Insulin ELISA (#80INSMR-CH01, Alpco).

### Human islets

Human islets from cadaveric, non-diabetic donors (donor characteristics in Supplemental Table 2) were supplied by the Alberta Diabetes Institute (Edmonton, Canada). Upon receipt, islets were hand-picked to 99% purity, and were cultured in CMRL (supplemented with 100 U/mL penicillin, 100 μg/mL streptomycin, 0.05 mg/mL Gentamicin, and 2 mmol/L glutamax) containing 11 mM glucose at 25 °C overnight prior to dispersion for FACS.

### Statistical analyses

With the exception of transcriptome analyses, all statistical analyses were performed using GraphPad Prism 8.0 (GraphPad Software, La Jolla, USA). Data were tested for normality by Shapiro-Wilk test. For comparisons of only two groups, normally distributed data were analyzed by t-test with Welch’s correction whereas non-normal data were analyzed by Mann-Whitney test. For comparisons of more than two groups, normally distributed data were analyzed by one-way ANOVA and non-normal data analyzed by Kruskal-Wallis test. For repeated measures of two or more groups (as in GTTs and ITTs) data were analyzed by two-way repeated measures ANOVA with Geisser-Greenhouse correction for non-sphericity. Data in all figures and text are represented as mean ± SD unless otherwise specified.

## Supplementary Information

**Additional file 1.**

## Data Availability

Open source data on each human islet preparation can be obtained at www.isletcore.ca. The flow cytometry datasets used and/or analysed during the current study are available from the corresponding author on reasonable request.

## Abbreviations

FSC: Forward-scatter
GTT: Glucose tolerance test
HFD: High fat diet
ITT: Insulin tolerance test
LFD: Low fat diet
SSC: Side-scatter
TCR: T cell receptor

## References

[1] Ehses JA, Perren A, Eppler E, Ribaux P, Pospisilik JA, Maor-Cahn R (2007). Increased number of islet-associated macrophages in type 2 diabetes. Diabetes..

[2] Butcher MJ, Hallinger D, Garcia E, Machida Y, Chakrabarti S, Nadler J (2014). Association of proinflammatory cytokines and islet resident leucocytes with islet dysfunction in type 2 diabetes. Diabetologia..

[3] Richardson SJ, Willcox A, Bone AJ, Foulis AK, Morgan NG (2009). Islet-associated macrophages in type 2 diabetes. Diabetologia..

[4] Westwell-Roper C, Dai DL, Soukhatcheva G, Potter KJ, van Rooijen N, Ehses JA (2011). IL-1 blockade attenuates islet amyloid polypeptide-induced proinflammatory cytokine release and pancreatic islet graft dysfunction. J Immunol.

[5] Masters SL, Dunne A, Subramanian SL, Hull RL, Tannahill GM, Sharp FA (2010). Activation of the NLRP3 inflammasome by islet amyloid polypeptide provides a mechanism for enhanced IL-1β in type 2 diabetes. Nat Immunol.

[6] Westwell-Roper CY, Ehses JA, Verchere CB (2014). Resident macrophages mediate islet amyloid polypeptide-induced islet IL-1β production and β-cell dysfunction. Diabetes..

[7] Meier DT, Morcos M, Samarasekera T, Zraika S, Hull RL, Kahn SE (2014). Islet amyloid formation is an important determinant for inducing islet inflammation in high-fat-fed human IAPP transgenic mice. Diabetologia..

[8] Westwell-Roper C, Denroche HC, Ehses JA, Verchere CB (2016). Differential activation of innate immune pathways by distinct islet amyloid polypeptide (IAPP) aggregates. J Biol Chem.

[9] Kamata K, Mizukami H, Inaba W, Tsuboi K, Tateishi Y, Yoshida T (2014). Islet amyloid with macrophage migration correlates with augmented β-cell deficits in type 2 diabetic patients. Amyloid..

[10] Nackiewicz D, Dan M, He W, Kim R, Salmi A, Rütti S (2014). TLR2/6 and TLR4-activated macrophages contribute to islet inflammation and impair beta cell insulin gene expression via IL-1 and IL-6. Diabetologia..

[11] Calderon B, Carrero JA, Ferris ST, Sojka DK, Moore L, Epelman S (2015). The pancreas anatomy conditions the origin and properties of resident macrophages. J Exp Med.

[12] Calderon B, Suri A, Miller MJ, Unanue ER (2008). Dendritic cells in islets of Langerhans constitutively present cell-derived peptides bound to their class II MHC molecules. Proc Natl Acad Sci.

[13] Ferris ST, Carrero JA, Mohan JF, Calderon B, Murphy KM, Unanue ER (2014). A minor subset of Batf3-dependent antigen-presenting cells in islets of langerhans is essential for the development of autoimmune diabetes. Immunity..

[14] Zinselmeyer BH, Vomund AN, Saunders BT, Johnson MW, Carrero JA, Unanue ER (2018). The resident macrophages in murine pancreatic islets are constantly probing their local environment, capturing beta cell granules and blood particles. Diabetologia..

[15] Dror E, Dalmas E, Meier DT, Wueest S, Thévenet J, Thienel C (2017). Postprandial macrophage-derived IL-1β stimulates insulin, and both synergistically promote glucose disposal and inflammation. Nat Immunol.

[16] Banaei-Bouchareb L, Gouon-Evans V, Samara-Boustani D, Castellotti MC, Czernichow P, Pollard JW (2004). Insulin cell mass is altered in Csf1op/Csf1op macrophage-deficient mice. J Leukoc Biol.

[17] Brissova M, Aamodt K, Brahmachary P, Prasad N, Hong JY, Dai C (2014). Islet microenvironment, modulated by vascular endothelial growth factor-a signaling, promotes beta cell regeneration. Cell Metab.

[18] Riley KG, Pasek RC, Maulis MF, Dunn JC, Bolus WR, Kendall PL (2015). Macrophages are essential for CTGF-mediated adult β-cell proliferation after injury. Mol Metab.

[19] Xiao X, Gaffar I, Guo P, Wiersch J, Fischbach S, Peirish L (2014). M2 macrophages promote beta-cell proliferation by up-regulation of SMAD7. Proc Natl Acad Sci U S A.

[20] Han JM, Wu D, Denroche HC, Yao Y, Verchere CB, Levings MK (2015). IL-33 reverses an obesity-induced deficit in visceral adipose tissue ST2 + T regulatory cells and ameliorates adipose tissue inflammation and insulin resistance. J Immunol.

[21] Feuerer M, Herrero L, Cipolletta D, Naaz A, Wong J, Nayer A (2009). Lean, but not obese, fat is enriched for a unique population of regulatory T cells that affect metabolic parameters. Nat Med.

[22] Eller K, Kirsch A, Wolf AM, Sopper S, Tagwerker A, Stanzl U (2011). Potential role of regulatory T cells in reversing obesity-linked insulin resistance and diabetic nephropathy. Diabetes..

[23] Winer S, Chan Y, Paltser G, Truong D, Tsui H, Bahrami J (2009). Normalization of obesity-associated insulin resistance through immunotherapy. Nat Med.

[24] Han JM, Patterson SJ, Speck M, Ehses JA, Levings MK (2014). Insulin inhibits IL-10–mediated regulatory T cell function: implications for obesity. J Immunol.

[25] Nishimura S, Manabe I, Takaki S, Nagasaki M, Otsu M, Yamashita H (2013). Adipose natural regulatory B cells negatively control adipose tissue inflammation. Cell Metab.

[26] Shen L, Chng MHY, Alonso MN, Yuan R, Winer DA, Engleman EG (2015). B-1a lymphocytes attenuate insulin resistance. Diabetes..

[27] Harmon DB, Srikakulapu P, Kaplan JL, Oldham SN, McSkimming C, Garmey JC (2016). Protective role for B-1b B cells and IgM in obesity-associated inflammation, glucose intolerance, and insulin resistance. Arterioscler Thromb Vasc Biol.

[28] Winer D, Winer S, Shen L, Wadia PP, Yantha J, Paltser G (2011). AB cells promote insulin resistance through modulation of T cells and production of pathogenic IgG antibodies. Nat Med.

[29] Bapat SP, Myoung Suh J, Fang S, Liu S, Zhang Y, Cheng A (2015). Depletion of fat-resident T reg cells prevents age-associated insulin resistance. Nature..

[30] Lumeng CN, Liu J, Geletka L, Delaney C, Delproposto J, Desai A (2011). Aging is associated with an increase in T cells and inflammatory macrophages in visceral adipose tissue. J Immunol.

[31] Krishna KB, Stefanovic-Racic M, Dedousis N, Sipula I, O’Doherty RM (2016). Similar degrees of obesity induced by diet or aging cause strikingly different immunologic and metabolic outcomes. Physiol Rep.

[32] Carter S, Miard S, Caron A, Sallé-Lefort S, St-Pierre P, Anhê FF (2018). Loss of OcaB prevents age-induced fat accretion and insulin resistance by altering B-lymphocyte transition and promoting energy expenditure. Diabetes..

[33] Ackert-Bicknell C, Beamer W, Rosen C, Sundberg J. Aging study: Bone mineral density and body composition of 32 inbred strains of mice. MPD: Ackert1. Mouse Phenome Database web resource (RRID:SCR_003212) The Jackson Laboratory, Bar Harbor, Maine USA.https://phenome.jax.org [cited (September 4, 2019)].

[34] Leiter EH, Premdas F, Harrison DE, Lipson LG (1988). Aging and glucose homeostasis in C57BL/6J male mice. FASEB J.

[35] Ehrhardt N, Cui J, Dagdeviren S, Saengnipanthkul S, Goodridge HS, Kim JK (2019). Adiposity-independent effects of aging on insulin sensitivity and clearance in mice and humans. Obesity..

[36] Helman A, Klochendler A, Azazmeh N, Gabai Y, Horwitz E, Anzi S (2016). p16 Ink4a-induced senescence of pancreatic beta cells enhances insulin secretion. Nat Med.

[37] Avrahami D, Li C, Zhang J, Schug J, Avrahami R, Rao S (2015). Aging-dependent demethylation of regulatory elements correlates with chromatin state and improved β cell function. Cell Metab.

[38] Ferris ST, Zakharov PN, Wan X, Calderon B, Artyomov MN, Unanue ER (2017). The islet-resident macrophage is in an inflammatory state and senses microbial products in blood the journal of experimental medicine. J Exp Med.

[39] Chen H, Eling N, Martinez-Jimenez CP, O’Brien LM, Carbonaro V, Marioni JC (2019). IL −7-dependent compositional changes within the γδ T cell pool in lymph nodes during ageing lead to an unbalanced anti-tumour response. EMBO Rep.

[40] Nackiewicz D, Dan M, Speck M, Chow SZ, Chen YC, Pospisilik JA (2019). Islet macrophages shift to a reparative state following pancreatic beta-cell death and are a major source of islet IGF-1. iScience.

[41] Radenkovic M, Uvebrant K, Skog O, Sarmiento L, Avartsson J, Storm P (2017). Characterization of resident lymphocytes in human pancreatic islets. Clin Exp Immunol.

[42] Dalmas E, Lehmann FM, Dror E, Wueest S, Thienel C, Borsigova M (2017). Interleukin-33-Activated Islet-Resident Innate Lymphoid Cells Promote Insulin Secretion through Myeloid Cell Retinoic Acid Production. Immunity.

[43] In’t Veld P, Lievens D, De Grijse J, Ling Z, Van Der Auwera B, Pipeleers-Marichal M (2007). Screening for insulitis in adult autoantibody-positive organ donors. Diabetes..

[44] Hajmrle C, Smith N, Spigelman AF, Dai X, Senior L, Bautista A (2016). Interleukin-1 signaling contributes to acute islet compensation. JCI Insight.

[45] Park SL, Zaid A, Hor JL, Christo SN, Prier JE, Davies B (2018). Local proliferation maintains a stable pool of tissue-resident memory T cells after antiviral recall responses. Nat Immunol.

[46] Bender C, Rodriguez-Calvo T, Amirian N, Coppieters KT, von Herrath MG (2020). The healthy exocrine pancreas contains preproinsulin-specific CD8 T cells that attack islets in type 1 diabetes. Sci Adv.

[47] Dirice E, Kahraman S, Jiang W, El Ouaamari A, De Jesus DF, Teo AKK (2014). Soluble factors secreted by T cells promote β-cell proliferation. Diabetes..

[48] Prata LGPL, Ovsyannikova IG, Tchkonia T, Kirkland JL (2018). Senescent cell clearance by the immune system: emerging therapeutic opportunities. Semin Immunol.

[49] Aguayo-Mazzucato C, Andle J, Lee TB, Midha A, Talemal L, Chipashvili V (2019). Acceleration of β cell aging determines diabetes and senolysis improves disease outcomes. Cell Metab.

[50] Thompson PJ, Shah A, Ntranos V, van Gool F, Atkinson M, Bhushan A (2019). Targeted Elimination of Senescent Beta Cells Prevents Type 1 Diabetes Article Targeted Elimination of Senescent Beta Cells Prevents Type 1 Diabetes. Cell Metab.

[51] Markle JGM, Mortin-Toth S, Wong ASL, Geng L, Hayday A, Danska JS (2013). γδ T cells are essential effectors of type 1 diabetes in the nonobese diabetic mouse model. J Immunol.

[52] Aguayo-Mazzucato C (2020). Functional changes in beta cells during ageing and senescence. Diabetologia..

