# Supplementary material for: T cells accumulate in non-diabetic islets during ageing
Source: Immun Ageing. 2021 Feb 23;18:8. doi: 10.1186/s12979-021-00221-4 (PMC7901217; doi:10.1186/s12979-021-00221-4)
Supplement: Supplementary file 1 — Additional file 1. [file 12979_2021_221_MOESM1_ESM.pdf]

Supplemental Table 1. Antibodies for flow cytometry

Target	Clone	Catalogue	Dilution
		**Mouse islets**	
**CD45**	30-F11	48-0451-82	1:100
**CD19**	eBio1D3	17-0193-82	1:100
**CD5**	53-7.3	11-0051-82	1:150
**CD11b**	M1/70	12-0112-82	1:100
**CD3**	17A2	564009	1:100
**CD4**	GK1.5	100453	1:100
**CD3e**	145-2C11	25-0031-82	1:25
**CD8a**	53-6.7	100761	1:100
**FoxP3**	FJK-16s	11-5773-82	1:25
**TCR b**	H57-597	109212	1:100
**TCR gd**	GL3	118108	1:100
**NK1.1**	PK136	108714	1:100
		**Human islets**	
**CD45**	HI30	48-0459-42	1:15
**CD3**	UCHT1	17-0038-42	1:20

Supplemental Table 2. Human islet donor characteristics.

Islet preparation	1	2	3
	**Donor demographics**		
**Unique identifier**	R310	R314	R317
**Age (years)**	25	31	54
**Sex (M/F)**	M	F	M
**BMI (kg/m^2)**	26.4	30.3	26.4
**HbA_{1c}**	5.4	5.0	5.1
**Cause of death**	NDD-neurological	NDD-neurological	NDD-neurological
**Diabetes? (Y/N)**	N	N	N
	**Pancreas**		
**Origin/source**	Alberta Diabetes Institute Islet Core	Alberta Diabetes Institute Islet Core	Alberta Diabetes Institute Islet Core
**Cold ischaemia time (h)**	11.25	14.75	21
	**Islet handling and use**		
**Origin/source**	Alberta Diabetes Institute Islet Core	Alberta Diabetes Institute Islet Core	Alberta Diabetes Institute Islet Core
**Isolation centre**	Alberta Diabetes Institute Islet Core	Alberta Diabetes Institute Islet Core	Alberta Diabetes Institute Islet Core
**Estimated purity (%)**	90	80	90
**Total culture time (h)**	~96	~48	~96
**Functional measurement (Stimulation index mean 10 mM to 1 mM)**	14.07	3.62	11.9
**Experimental islet use (including in which experiment each islet preparation was used)**	Islet dispersion and flow cytometry	Islet dispersion and flow cytometry	Islet dispersion and flow cytometry

This table was prepared following recommendations as per: Hart N.J., Powers A.C. 2019. Use of human islets to understand islet biology and diabetes: progress, challenges and suggestions. Diabetologia 62:212–222. Open source data on each islet preparation can be obtained at www.isletcore.ca.

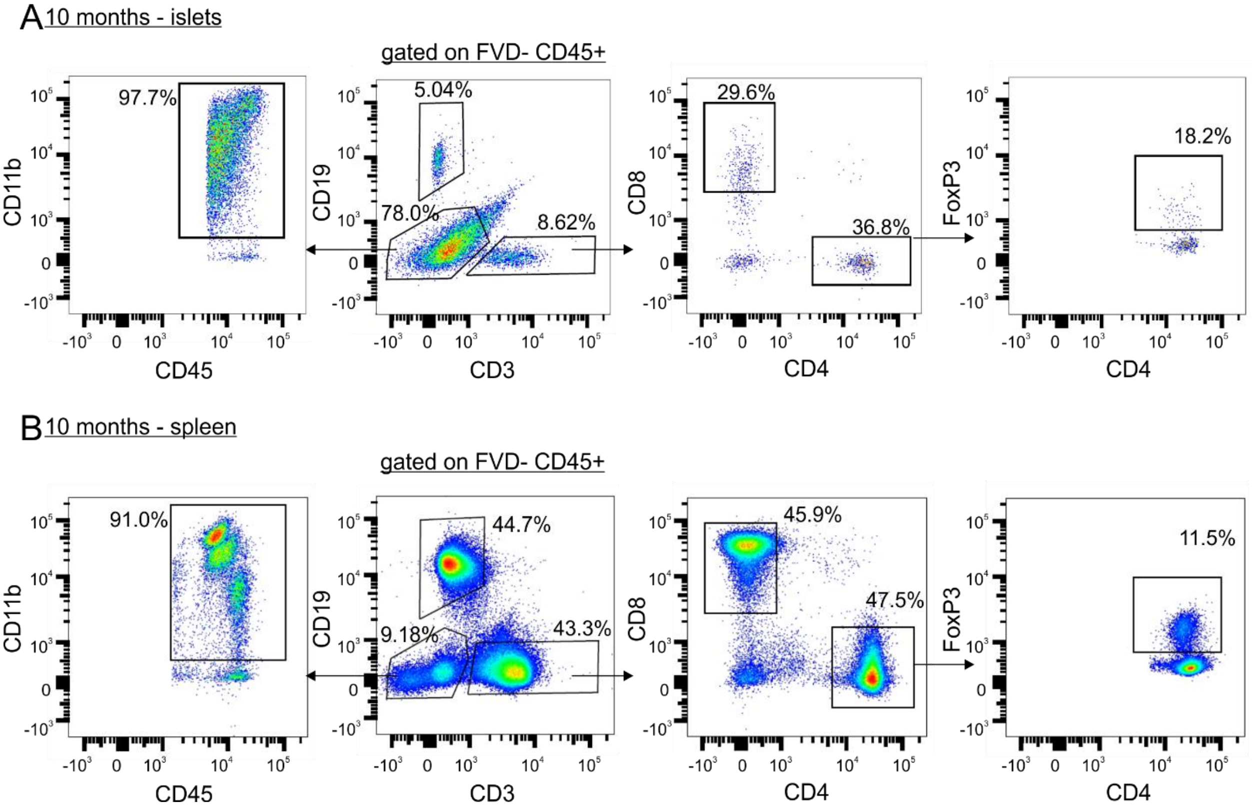

Supplemental Figure 1. T cell subsets in 10 month-old mice. Islets (A) and spleens (B) were isolated from 10-month old mice for analysis of islet T cell subsets by flow cytometry. Cells were gated on FSC, SSC, viability (FVD-) and CD45+, and subsequently CD19, CD3, CD11b, CD8, CD4, and FoxP3; data are representative of 2 samples, (for islet samples, islets from 10 mice pooled per sample).

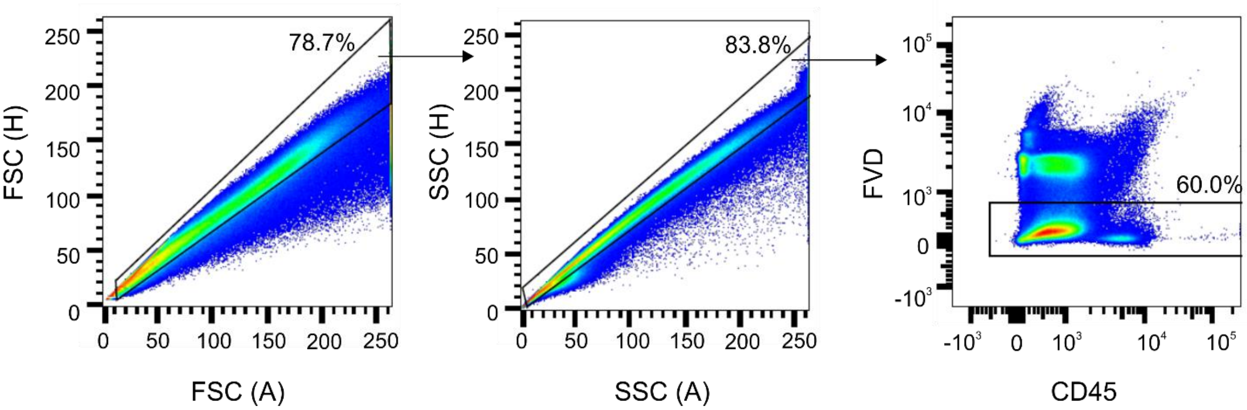

Supplemental Figure 2. Representative data from human islet preparations, dispersed and gated on FSC, SSC and viability (FVD-). FACS plots are representative of 3 independent biological samples. Numbers in FACS plots represent the percent of cells in each selection as a function of the parent population.
